# Efficacy of neurofeedback in the treatment of Dyslexia: a systematic review

**DOI:** 10.1007/s11881-025-00335-0

**Published:** 2025-07-17

**Authors:** Miguel López-Zamora, Nadia Porcar-Gozalbo, María Rodríguez Moreno, Alejandro Cano-Villagrasa, Laura Bandera Pastor

**Affiliations:** 1 Universidad de Málaga, Málaga, España; 2 Universidad Internacional de Valencia, Valencia, España; 3 Universidad de Málaga, Málaga, España; 4 Universidad Internacional de Valencia, Valencia, España; 5 Málaga, España

**Keywords:** Biofeedback, Dyslexia, EEG, Neurofeedback

## Abstract

Dyslexia is a neurodevelopmental disorder that impairs the learning and proficiency of reading and writing processes, persisting throughout the individual's life. Most interventions are educational in nature, but studies published over the last decade suggest that Neurofeedback may serve as a substantial complement to improving reading skills. The primary objective was to analyze the efficacy of various protocols used within Neurofeedback techniques for the treatment of reading impairments. A total of 12 research articles related to Neurofeedback treatment in populations with reading process impairments were selected. The PRISMA guidelines were followed in conducting this study. The inclusion criteria were: (I) Participants diagnosed with dyslexia, (II) Neurofeedback-based interventions, (III) Articles written in Spanish or English, and (IV) Articles published within the last 20 years. Based on these criteria, a total of 12 research articles related to Neurofeedback treatment in populations with reading process impairments were selected. The results from the selected research articles revealed that Neurofeedback techniques alone do not present a significant benefit in improving reading skills in the dyslexic population. Additionally, the heterogeneity of research designs in the articles addressing this topic was highlighted, which complicated the determination of an unbiased criterion regarding the efficacy of such interventions. Currently, there is no significant evidence to determine the efficacy of Neurofeedback interventions in the dyslexic population. Further refinement of intervention methods and the development of cutting-edge technology are necessary to increase evidence regarding the effectiveness of these treatments for dyslexia.

**Supplementary Information:**

The online version contains supplementary material available at 10.1007/s11881-025-00335-0.

## Introduction

Developmental Dyslexia (DD) is defined as a neurobiological disorder characterized by a set of phonologically-based difficulties that hinder the proper acquisition of reading skills and persist throughout an individual's development. These difficulties are not a result of intellectual deficits, sensory dysfunction, socioeconomic disadvantage, or lack of educational opportunities (APA, 2013; Snowling & Hulme, 2012). In DD, several characteristic cognitive impairments have been described, including deficits in phonological awareness, short-term verbal memory, visual-auditory association, and rapid naming (Lyon et al., 2003).

Current neuroimaging techniques have shown a correlation between these cognitive impairments and the presence of specific brain differences in DD relative to typical readers. Functional magnetic resonance imaging (fMRI) has identified crucial brain circuits involved in the reading process in both neurotypical individuals and those with DD (Cuetos et al., 2019; Hancock et al., 2017; Martin et al., 2016; Richlan, 2020). These findings have contributed to the development of a neuroanatomical model that identifies lower activation in readers with dyslexia compared to typical readers. Reduced activity has been consistently observed in the left posterior temporoparietal cortex (including the middle and superior temporal gyri, supramarginal gyrus, and angular gyrus), the left occipitotemporal cortex (including the inferior temporal and fusiform gyri), and the left frontal cortex (including the inferior frontal and precentral gyri). Similarly, electroencephalography (EEG) studies have shown that the activity of certain frequency bands, as well as the interaction between these bands, plays a crucial role in various cognitive tasks involved in reading and writing (Hämäläinen et al., 2013). Specifically, EEG studies have explored the brain activation patterns in individuals with dyslexia (Arns et al., 2014; Goswami, 2018; Penolazzi et al., 2008), revealing that one of the main differences compared to typical readers is a greater activation and bilateral correlation of slow frequencies (Delta and Theta) in the left frontal and right temporal regions. Additionally, the increased activation of the right hemisphere in individuals with DD during reading tasks has been interpreted as a compensatory brain strategy (Shaywitz & Shaywitz, 2005), which, in turn, manifests with an increase in spectral coherence and activation of the fast Alpha and Beta bands (Arns et al., 2014; Penolazzi et al., 2008). Furthermore, in children with DD, greater activation of Delta waves has been found in the left frontal area during phonological tasks (Penolazzi et al., 2008), which is associated with alterations in the execution of cortical inhibitory processes.

Despite the abundant evidence of anomalies in brain activation in individuals with DD, this knowledge is often not considered in their intervention programs. Most intervention methods aimed at overcoming difficulties related to DD, as well as the measures used to evaluate the efficacy of the intervention, are primarily behavioral and include activities aimed at improving reading and writing skills through the adoption of cognitive or educational process-based approaches (Gaab et al., 2007; Saine et al., 2011; Shaywitz et al., 2004; Thomson et al., 2013). These interventions involve explicit instruction and address the behavioral components of reading: phonological awareness, phonics, fluency, vocabulary, and comprehension. From these studies, individual non-responsiveness to intervention has been correlated with low skills in rapid naming, phonological awareness, alphabetic principle, and verbal memory (Nelson et al., 2003).

While numerous studies have examined the effectiveness of behavioral interventions and their cognitive-behavioral outcomes, far fewer have investigated the neurobiological effects that such interventions may induce. This is a critical gap, as understanding whether reading-based treatments produce measurable changes in brain activation is essential both for validating their theoretical underpinnings and for refining their application. In this regard, a meta-analysis conducted by Barquero et al. (2014) reviewed 14 functional neuroimaging studies and identified consistent activation changes following reading interventions, particularly in regions such as the left thalamus, right inferior frontal insula, left inferior frontal cortex, right posterior cingulate, and left middle occipital gyrus. These findings demonstrate the potential of behavioral interventions to modulate brain function and underscore the importance of incorporating neurobiological measures in treatment evaluation.

The initial description of the term Biofeedback (BF) first appeared in case studies conducted by Lubar and Shouse (1976), in which operant conditioning techniques were used to reinforce specific types of electrophysiological activity to treat the core symptoms of attention deficit hyperactivity disorder (ADHD). Through visual and auditory feedback for certain neural behaviors, a reduction in hyperactive behavior and an improvement in attention were observed after decreasing cortical activity in the theta frequencies. This study was one of the pioneers in the use of electrophysiological techniques to measure, process, and intervene in bodily functions with the aim of helping individuals to self-regulate them more effectively. Following these studies, there has been a growing body of research advocating the application of BF techniques for the rehabilitation of various disorders.

Among BF treatments, the most commonly used is Neurofeedback (NF) (Coben & Evans, 2010). NF represents a specific form of BF that utilizes an electroencephalograph and is therefore also referred to as EEG-biofeedback (Carrobles, 2016). In a typical neurofeedback session, participants may observe visual feedback, such as a moving bar, a rocket, or animated shapes, on a screen that changes in real time based on their brain activity. This visual cue informs the participant when their brain is producing the desired neural patterns, reinforcing these patterns through repetition. This technique operates by collecting EEG waves through its electrodes, transforming them into visual or tactile signals that serve as feedback for the behavior targeted for intervention (Collura et al. 2014). Depending on the rhythm and frequency (Carrobles, 2016), NF can be applied to modify specific psychological states, particularly by using connectivity-based NF techniques (Bassett & Sporns, 2017). Connectivity-based NF relies on the existence of links between interregional brain connectivity and psychological disorders (Whitfield-Gabrieli & Ford, 2012). This technique can be used to design various clinical interventions that facilitate the reinforcement of direct and unconscious brain activity in the human brain, as well as improve metacognition and the ability to perceive one's own brain activity (Cortese et al., 2016; TaschereAu-Dumouchel et al., 2022). In this regard, NF has shown effectiveness in numerous disorders such as learning difficulties (Olulade et al., 2013), ADHD (Au et al., 2014; MicoulAud-Franchi et al., 2014), epilepsy (Sterman & Egner, 2006), autism (García-Berjillos et al., 2015), depression (KAur et al., 2019), schizophrenia (Surmeli et al., 2012), post-traumatic stress disorder (Askovic et al., 2023), and dyslexia (Marzbani et al., 2016). In a recent meta-analysis (TaschereAu-Dumouchel et al., 2022), which conducted an extensive review using a very strict selection method on the efficacy of NF interventions for the rehabilitation of specific psychological disorders (depressive disorders, anxiety disorders, substance abuse, ADHD, autism spectrum disorder, post-traumatic stress disorder, and schizophrenia), mixed but promising results were found. Specifically, Taschereau-Dumouchel et al. (2022) concluded that, currently, NF applications could be considered"probably effective"psychological treatments, with interventions for major depressive disorder showing the most empirical support. However, this research warns that some NF treatment strategies still do not meet the criteria for well-established treatments, lacking methodological rigor, primarily due to inadequately describing the theoretical models they are based on, as well as evaluations of the intervention programs. For this reason, the efficacy of NF has not been demonstrated in many studies.

One area where NF appears to offer potential benefits is in children with learning difficulties, particularly in processes related to reading and writing, such as attention, working memory, and more broadly, intelligence and academic performance (Fernández et al., 2007a, 2007b; Mosanezhad Jeddi & Nazari, 2013). Unlike clinical disorders, NF treatments aimed at improving reading and writing processes associated with specific learning disorders, such as dyslexia and dysgraphia have been studied to a lesser extent, despite this condition presenting clear and well-studied neuroanatomical anomalies (Ramus et al., 2018). To examine the promising implications that NF treatment may have in the rehabilitation of the core symptoms of dyslexia, this study proposes a systematic review of the studies conducted in this field.

Thus, this study proposes, on one hand, to analyze and describe all studies that have carried out NF treatment to address symptoms affecting the proper performance of reading and writing, and on the other hand, to identify methodological strengths and weaknesses of the treatments, with the ultimate goal of generating an initial framework that will serve future research to improve and design more sophisticated NF treatments with greater empirical validity.

## Method

### Study design

A systematic literature review was conducted following the guidelines and recommendations of the PRISMA model (Page et al., 2021). This review was guided by the following research question: What is the current evidence regarding the efficacy of neurofeedback-based interventions in improving reading-related difficulties in children diagnosed with dyslexia or related reading disorders?

### Eligibility criteria

For the purposes of this review, studies were selected based on the following inclusion criteria: (a) they employed an empirical design (experimental, quasi-experimental, or pre-post intervention); (b) they included children or adolescents formally diagnosed with dyslexia, developmental dyslexia, or a specific learning disorder affecting reading or spelling, as defined by DSM or ICD criteria; (c) the intervention was based on neurofeedback (NF), delivered through EEG or equivalent neurophysiological recording systems; and (d) they reported outcome measures related to reading performance, phonological awareness, attention, working memory, or academic achievement. Only studies published in peer-reviewed journals and written in English or Spanish were considered.

Exclusion criteria included: (a) theoretical papers, literature reviews, meta-analyses, conference abstracts, or editorials; (b) studies involving neurofeedback protocols unrelated to reading or cognitive outcomes (e.g., used exclusively for emotional regulation or motor control); (c) samples composed exclusively of individuals with neurological, psychiatric, or sensory disorders not comorbid with dyslexia (e.g., autism, epilepsy, intellectual disability); and (d) insufficient methodological detail to extract relevant data on participants, intervention procedures, or outcomes.

### Types of studies

The selected studies include experimental and quasi-experimental research, in which a pre-post test design was established (75%), randomized comparative design (12.5%), and single-case studies (12.5%).

### Types of participants

The participants included in the selected studies for this systematic review were children aged between 6 and 12 years (M = 9.7). All participants were diagnosed with Specific Learning Disorder in one of its forms (dyslexia and/or dysgraphia) according to the criteria established in the DSM-5 (APA, 2013).

### Study selection and data collection process

The literature search for this systematic review was conducted between September and November 2023 following PRISMA 2020 guidelines (Page et al., 2021). Electronic searches were performed in the following databases: PubMed, Medline, PsycINFO, and Web of Science. Additionally, a manual search was carried out using Google Scholar to identify relevant recent publications not captured in the initial database queries. The search strategy applied Boolean combinations of the terms *“Neurofeedback” AND “Dyslexia”*, used uniformly across all platforms. These terms were searched within article titles, abstracts, and keywords. No filters were applied during the initial search phase to maximize the sensitivity and comprehensiveness of the results.

To ensure relevance and methodological rigor, the search was limited to peer-reviewed journal articles written in English or Spanish and published within the last 20 years. Prior to initiating the review, inclusion and exclusion criteria were clearly defined in accordance with the objectives of the study. Furthermore, the search strategy was peer-reviewed using the PRESS checklist (Relevo & Paynter, 2012) to enhance transparency and quality.

### Risk of bias in individual studies

To assess the risk of bias in the individual studies included in this systematic review, the CASP checklist (Quigley et al., 2019) was used. This scale is based on three questions to determine the presence of biases in the studies: (A) Are the results of the study valid?; (B) What are the results?; (C) Will the results help locally? Additionally, to minimize the risk of bias in the selection of studies included in this review, two reviewers independently evaluated the titles and abstracts of the identified articles. Any discrepancies in the inclusion or exclusion of a study were discussed and resolved by consensus between the reviewers, and, if necessary, a third reviewer was consulted for the final decision. This rigorous approach ensures the integrity and impartiality of the study selection process. After applying these two measures, it was determined that there was a low risk of bias in the selected studies for this systematic review.

### Data extraction process

The guidelines and recommendations established in the PRISMA model were followed for the study selection and data extraction process. A detailed summary of the criteria and their correspondence to specific sections of the manuscript is available in Supplementary Table S1.

## Results

### Study selection

**Fig. 1:**
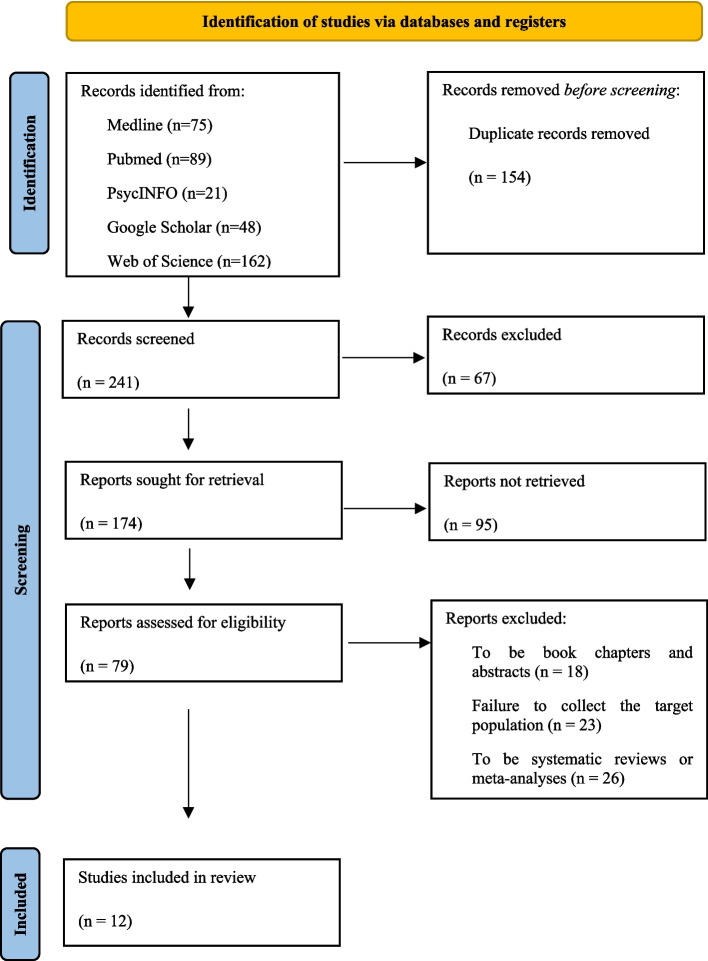
Modified PRISMA diagram of studies included in the systematic review

During the initial search for studies, a total of 395 potentially eligible articles were found, distributed as follows: 75 from Medline, 89 from PubMed, 21 from PsycINFO, 48 from Google Scholar (through a manual literature search), and 162 from Web of Science. The study selection process was carried out following the guidelines and recommendations of the PRISMA statement, as shown in Fig. 1. In total, 383 articles were excluded, with 12 articles selected for final inclusion in this systematic review. The most important aspects of the articles selected for this systematic literature review can be seen in Table 1.

**Table 1:** Summary of the empirical articles included in the systematic review

Author	Design	Sample	Protocol	Schedule	Evaluations	Results
Au et al. (2014)	Multiple baseline. Pre-post. No control group	3 Chinese children with dyslexia and 1 comorbid with ADHD, ages 9–12	Reduction of Theta rhythm and increase of Beta rhythm (θ/β protocol at C3 and C4)	10 sessions, 1/week, ~ 30–40 min	Intelligence (WISC-IV); attention (TEA-Ch); vigilance task (CPT); motor impulsivity (Stop-IT); phonological awareness (HKT-SpLD)	Improvement in visual and auditory attention, phonological awareness, and inhibitory control
Breteler et al., (2010)	Pre-post. Randomized controlled study. Control group	19 participants with dyslexia. Mean age: 10.33 years	Different protocols: increase Delta activity at T6, increase Alpha or Beta coherence at F7-FC3, or increase coherence at T3-74 with Z	21 sessions, 3/week, 45 min	Language test (rapid letter reading, articulation, phoneme elimination, and spelling), EEG, and NP tests (memory: retrieval and recognition; interference; dominant hand; speed, sustained, and divided attention)	Significant improvement in spelling. No evidence of efficacy in reading skills
Fernández et al., (2007a, 2007b)	Pre-post. Control group	16 subjects with learning difficulties: 11 in experimental group, 5 in control group. Ages: 7–11 years	Theta-Alpha protocol. Intermittent reinforcement	20 sessions, ~ 2/week, 30 min	TOVA, EEG, WISC-R, and parent interview	Immediate behavioral and cognitive improvements, reflected in EEG changes
Fernández et al. (2016)	Pre-post. No control group	20 children with learning difficulties (ages 6–12)	Theta-Alpha protocol	20 sessions, 3/week, 40 min	TOVA, EEG, WISC-R, and parent interview	Auditory reinforcement is more effective in Theta-Alpha regulation
Harandi and Moghadam (2017)	Pre-post. Randomization. No control group	26 subjects with dysgraphia (ages 7–11). 2 groups: NF and Fernald approach	Group 1: Increase SMR at C3 and C4). Group 2: Multisensory Fernald treatment	15 sessions, 3/week, ~ 30 min	Dictation evaluation test and Raven's Progressive Matrices	Both methods are effective in improving dictation performance. NF showed a greater effect size
Mehran et al. (2016)	Pre-post. Control group	6 participants with dyslexia. Ages: 7–10 years	Combined ELF and NF treatment. Reduce Delta and Theta and increase Beta at F3	6 sessions, 1/week, 30 min	Auditory Continuous Performance Test (IVA/CPT), Atieh Standardized Questionnaire, and EEG	Reduction in Theta rhythm and increase in IVA test performance. Relative improvement in reading ability
Mosanezhad Jeddi and Nazari (2013)	Pre-post. Single case. Multiple baselines	6 boys with dyslexia (ages 8–10)	Reduce Delta and Theta and increase Beta at T3 and F7	20 sessions, 2–3/week, 30 min	Continuous performance tasks, digit span subscale of WISC-III, and EEG	Improvements in attention, working memory, and coherence in Theta, Delta, and Beta
Nazari et al., (2012)	Single case design. Multiple baselines. Pre-post. Randomization	6 boys aged 8 to 10 years with dyslexia	Decrease Delta and Theta, increase Beta at T3 and F7	20 sessions, 2–3/week, 30 min	Reading ability test (number of errors and reading length), phonological awareness test, and EEG	Significant improvement in reading and phonological awareness. Normalization of Theta band coherence at T3-T4, Delta, and Beta
Raesi et al., (2016)	A-B-A. No control group	4 children with dyslexia, aged 8–12 years	NF training: reduce Theta and Delta waves at F7 and increase Beta waves at T3	20 sessions, 3/week, 30 min	The standard text of name reading test (accuracy, speed, reading comprehension, and spelling)	Improvement in spelling, accuracy, and reading comprehension. Increased reading speed may require more sessions
Sadeghi and Nazari (2015)	A-B-A single case design. Pre-post	2 children aged 10–12 years with dyslexia	Theta-Beta protocol	12 sessions, 2/week, 30 min	Posner paradigm, EEG, and event-related potentials (ERP)	Improvement in processing speed and visuospatial attention
Walker and Norman (2006)	Pre-post	12 children with dyslexia	Protocols based on altered patterns	30–35 sessions, 3–4/week, 10 min	EEG analysis (two conditions: eyes open and closed), reading ability tests	Improvement of at least two grade levels in reading ability
Walker (2012)	Pre-post	24 subjects with treatment-refractory dysgraphia	5–10 NF sessions: decrease excessively slow or fast activity in the left frontal or central areas	8 sessions, 1–2/week, 20 min	Written expression checklists (page, sentence, and letter spacing)	Consistent improvement in dysgraphia refractory to other treatments

In relation to the methodological design of the 12 articles selected for this systematic review, quasi-experimental studies were primarily identified. These studies employed assessments at different points in time, both cross-sectional and longitudinal. Intra-group and inter-group analyses were conducted to evaluate the participants'data. This methodological approach in the selected studies provides a comprehensive and diverse view of NF intervention in the pediatric population with dyslexia. Additionally, a summary of the articles selected for this systematic review can be found in the following table (Table 1). Although some of the included studies refer to their samples using broader terms such as *learning disabilities* or *learning difficulties*, all of them focus on difficulties specifically related to reading processes. In these cases, the symptomatology described, the outcome measures selected (e.g., reading speed, spelling accuracy, phonological awareness), and the structure of the neurofeedback interventions clearly target deficits that fall within the scope of developmental dyslexia. Therefore, despite the variability in terminology, the studies meet the inclusion criteria of this review, as they all address core components of dyslexia either explicitly or through their intervention goals and measured outcomes.

The outcome measures assessed across studies included a range of standardized and experimental tasks targeting key domains associated with reading development. These comprised tests of reading accuracy, fluency, and comprehension; phonological awareness tasks such as phoneme segmentation, blending, and manipulation; attention measures including Continuous Performance Tests (e.g., CPT, TOVA); working memory tasks such as digit span; and general academic performance indicators including spelling tasks, dictation, and standardized achievement tests.

### Study quality

To assess the quality of the empirical studies included in this systematic review, the Axis Quality Assessment Score questionnaire (Downes et al., 2016) was used. The results indicated that the selected articles met the criteria outlined in the questionnaire.

### Analysis of the studies

A total of 12 empirical articles were identified during the review, and these were classified based on the NF protocol applied. This selection allows for a comprehensive analysis of the application of this technique, as well as a specific study of the effectiveness of each protocol.

### Protocol for reducing theta and/or delta and increasing beta

This protocol implements NF with the aim of reducing the activity of slow Theta waves (attention problems) and Delta waves (inhibition) while increasing fast Beta waves (concentration). Its use has been reported in 8 studies, with varying results (Au et al., 2014; Breteler et al., 2010; Fernández et al., 2007a, 2007b; Mehran et al., 2016; Mosanezhad Jeddi & Nazari, 2013; Nazari et al., 2012; Penolazzi et al., 2008; Raesi et al., 2016).

The research group led by Nazari at the University of Iran has been one of the most active in using this protocol, conducting three studies to date. In the first study (Nazari et al., 2012), they conducted 20 sessions with a program to reduce Delta-Theta bands and increase Beta in children with DD. The intervention significantly improved reading and performance on phonological awareness tests, and interhemispheric spectral coherence improvements were reported between central parietal and frontal areas. This was interpreted as more efficient integration of sensory and motor areas, which would explain the observed improvements in reading and phonology. Subsequently, they studied whether this protocol could improve other areas in children with DD (Mosanezhad Jeddi & Nazari, 2013), finding improvements in working memory and attention. In the third study (Sadeghi & Nazari, 2015), they successfully demonstrated how a Theta and Beta protocol could improve visuospatial attention in two children with dyslexia.

Other groups have replicated these findings. For example, the study by Raesi et al. (2016) applied this same protocol to 4 children with DD, finding significant improvements in reading accuracy, spelling, and comprehension. The authors suggested that the reduction of Theta and Delta waves improved phonological processing and working memory, while the increase in Beta improved attentional processes. Another pilot study (Au et al., 2014), this time using the Theta-Beta protocol, found improvements in 3 Chinese children with dyslexia and 1 comorbid with ADHD, showing that all improved in sustained and selective attention, attentional shift, and phonological awareness, with the comorbid subject showing the greatest improvement.

However, the application of neurofeedback protocols remains inconsistent across studies. Breteler et al. (2010), for instance, implemented a modified protocol aimed at reducing Delta waves while increasing Beta and Alpha activity to assess its impact on reading performance in children with dyslexia. The intervention group was compared with a control group of children with dyslexia who did not receive NF treatment. Results showed an improvement in spelling, but no significant gains in reading performance were observed. The authors attributed these findings to limitations in sample selection and the lack of control over important variables, such as whether participants were concurrently receiving other reading or writing interventions.

Other studies focused on exploring the combined effect of different types of interventions in DD, such as comparing the efficacy of this NF protocol with a very low-frequency magnetic stimulation program (Mehran et al., 2016). By exposing the right frontal hemisphere neurons of 6 children with dyslexia to very weak local magnetic fields, the authors aimed to improve their attentional capacities. The subjects showed improvements in these areas, but the results were inconclusive, as the effectiveness of each treatment could not be evaluated separately.

### Protocol for reducing theta and increasing alpha

Research using protocols for reducing Theta and increasing Alpha, focused on decreasing attention problems and enhancing self-regulation in DD, is not abundant. One notable study (Fernández et al., 2007a, 2007b) involved 6 children with learning difficulties, where all participants showed improved intelligence and attention scores. This study showed that the Theta/Alpha wave ratio became much more regulated during NF sessions, but these results only persisted two months after treatment in one-third of the sample. In a second study on DD (Fernández et al., 2016), the same group attempted to optimize the duration of this protocol by applying different reinforcers simultaneously, both auditory and visual, and compared the effectiveness of both techniques. The authors found that while both were effective in regulating attention and increasing Alpha rhythm, it was the auditory reinforcement that significantly improved performance in activation tasks and also in verbal intelligence.

Unfortunately, these studies have only been applied to improve general cognitive functioning, so the direct benefit of a Theta-Alpha protocol on the reading difficulties of DD remains to be studied.

### Sensorimotor protocol (SMR)

SMR protocols, which use the low Beta frequency, are based on the activation of the sensorimotor cortex to regulate concentration and movement control, and have been successfully applied to improve some tasks related to reading and writing. It has been shown (Harandi & Moghadam, 2017) that SMR is effective compared to other treatments. Involving 36 children with dysgraphia, they divided the sample into two groups, applying an SMR protocol to one and a visual stimulation treatment to the other. The results showed that the group receiving NF showed significant improvement in dictation tasks.

Moreover, SMR protocols have been applied in other studies that implement interventions in reading and writing while considering other variables. One of the earliest works was the single-case study by Breteler et al. (2010), which combined an SMR protocol with an electromyography treatment for a child with ADHD, dyslexia, and ocular instability. The intervention was associated with improvements in attention and hyperactivity-related symptoms. Although gains in reading performance were also reported, the authors did not offer a clear explanation for the underlying cause of these improvements.

### Protocols based on individual EEG activation patterns

EEG activation protocols function based on the individualized regulation of each subject's altered EEG. Depending on the specific analysis of each patient's electroencephalographic patterns, each protocol is designed specifically to match their characteristics. This review found two studies that used this technique. The first was the work of Walker and Norman (2006), which claims that they were able to improve reading speed and comprehension in 12 children with dyslexia by applying individualized NF protocols tailored to the specific characteristics of each child. In another similar study, Walker (2012) applied an NF protocol to a group of 24 subjects with dysgraphia aimed at adjusting activity in the central and frontal areas of the left hemisphere. The author found that his participants exhibited abnormalities in these cortical areas specialized in the motor control of writing, either due to excessively slow or overly fast waves, and applied the corresponding corrective NF protocol to the altered frequency. After ten sessions, all participants improved their scores on five markers evaluating written expression.

## Discussion

The present research aimed to conduct a systematic review of the effectiveness of Neurofeedback (NF) interventions in individuals presenting core symptoms of dyslexia. Despite certain limitations, such as the inclusion of all studies found that address the topic or the absence of quantitative analysis, this study offers a broad and updated review of the current state of research on the subject. To our knowledge, this is the first time a review of NF in this type of population has been presented. The results of this study confirm that there is some evidence supporting the idea that NF therapy, in combination with other types of therapy, can help improve reading-related skills in individuals with dyslexia. However, the results are highly heterogeneous, and conclusions regarding its effectiveness are not definitive. The results are discussed below.

From the articles reviewed, the most frequently used protocol was one that reduces slow waves (Theta/Delta) and increases Beta activity to address difficulties experienced by children with developmental dyslexia. Most of these studies reported direct improvements in reading accuracy, phonological awareness, spelling, and comprehension. Additionally, several showed positive effects on broader cognitive domains involved in reading, such as inhibitory control, working memory, and attention, as well as improvements in interhemispheric coherence and, in some cases, anxiety levels. Findings from other protocols were more mixed. Two studies applying Theta–Alpha training in children with DD found improvements primarily in attention-related processes, although in one of them, these gains did not persist beyond two months (Fernández et al., 2007a, 2007b). By contrast, studies applying SMR-based protocols reported more consistent results. Among them, the study by Harandi and Moghadam (2017), found significant improvements in dictation performance in children with dysgraphia. In addition, other studies not included in the final selection are relevant for contextualizing the effects of SMR protocols. For example, Barnea et al. (2005) evaluated lexical decision performance in typical readers, and Kouhbanani et al. (2015) examined phonological awareness in children with cochlear implants. These findings suggest that SMR-based neurofeedback may have potential benefits across a range of populations with language-related difficulties.

Finally, the use of NF protocols different from those mentioned above, such as those applied based on individual EEG activity, was analyzed. Of the two studies reviewed, one with children with dyslexia (Walker & Norman, 2006) and another with dysgraphia (Walker, 2012), both, respectively, were associated with improved reading and writing skills of the children who received the intervention. Another different technique is the use of fMRI to apply NF, and although the reviewed study (RobineAu et al., 2014) did not use the technique with children with DD, its effectiveness in regulating interhemispheric balance in attentional tasks may be of interest if applied to participants with DD.

After conducting the review, we can conclude that the protocol for reducing slow waves (Theta/Delta) and increasing Beta is the most employed among the analyzed studies, providing favorable results in 77% of cases. However, these interventions are not without limitations. Some of the presented studies have small sample sizes (Au et al., 2014; Mehran et al., 2016; Mosanezhad Jeddi & Nazari, 2013; Nazari et al., 2012; Penolazzi et al., 2008; Raesi et al., 2016; Sadeghi & Nazari, 2015), very brief treatments (Au et al., 2014), non-generalizable results, or limitations in follow-up (Mehran et al., 2016). However, all the articles highlight that NF therapy alone does not improve reading processes in the population with dyslexia. The results of these interventions are positive only when directly combined with speech therapy or psychological therapy focused on aspects related to reading processes. Therefore, one of the key aspects of NF therapies is that they serve as a complement to direct therapy focused on improving these dimensions of reading, such as lexical access, reading speed, or attention, among others.

Similarly, it is important to highlight the importance of precise and thorough diagnosis when addressing dyslexia in children, especially when considering therapies like NF. Although this technique has gained interest as a possible therapeutic approach to improving brain functions related to dyslexia, its effectiveness is hindered by the complex and heterogeneous nature of this condition (Gevensleben et al., 2012). One of the main barriers lies in the associated comorbidities, which are common among children with dyslexia (Peterson & Pennington 2012). These comorbidities, which can include language development disorders, attention deficits, and even autism spectrum disorders (Peterson & Pennington 2012), share overlapping symptoms and affect different areas of cognitive functioning (Reid, 2019). Consequently, finding a single therapeutic approach that effectively addresses all these facets is extremely challenging. NF, by focusing on modulating brain activity, may not be specific enough to meet the individual needs of each child, especially when multiple conditions are present (Sonuga-Barke et al., 2014). Additionally, the lack of uniformity in neurofeedback protocols and the variability in children's responses to this therapy also contribute to its limited utility in treating dyslexia and its comorbidities (Gevensleben et al., 2012). Instead of relying solely on neurofeedback, a comprehensive approach that includes educational interventions, language therapies, and support strategies tailored to the individual needs of each child may be more beneficial in addressing the complexity of dyslexia and its concomitant manifestations (Peterson & Pennington 2012).

On the other hand, it is necessary to emphasize that, although a large number of reviewed studies propose interventions in patients'reading and writing skills, few studies directly intervene in phonological awareness, the root of dyslexia and the main target of the most successful interventions that use other methodologies (Harandi & Moghadam, 2017). Of the studies reviewed, only 4 directly focus on improving phonological awareness, while the remaining 8 focus on general skills such as intelligence or attention. It is also noteworthy that a high number of studies report improvements specifically in the spelling processes of individuals with DD, such as in dictation tasks, lexical decision, or reading speed.

In our view, this reflects a lack of theoretical focus in the application of NF to dyslexia and underscores the need to determine whether such interventions can directly impact the core components of reading. Furthermore, as highlighted by Barquero et al. (2014), even studies focusing on behavioral reading interventions face substantial methodological limitations that complicate the interpretation of neuroplastic changes. These include high heterogeneity in intervention types, neuroimaging tasks, and participant characteristics; small sample sizes; lack of adequate control groups; and inconsistent replication of specific activation patterns. Importantly, many of these studies report neural changes without establishing clear correlations with behavioral improvements in reading, which makes causal inference difficult.

Similarly, Perdue et al. (2022), in a recent systematic review and meta-analysis, found no consistent effects in brain activation changes following reading interventions and emphasized the variability across study designs, samples, and analytic methods. They advocate for a more integrated framework that accounts for interactions across distributed cognitive, linguistic, and sensory systems rather than simplistic models of normalization or compensation. These limitations underscore the necessity for more rigorous, replicable, and well-powered studies to establish a reliable link between intervention and brain function in reading disorders. Added to this are the mixed results, insufficient evidence, articles published in journals of questionable scientific quality, and, in several investigations, serious methodological shortcomings. This led us to exclude several published articles from the review that, upon closer examination, did not meet the methodological standards required by the inclusion criteria established for the consulted databases.

In summary, the body of studies on NF applied to populations with DD is limited, showing deficiencies in its theoretical framework and methodological rigor, as well as a small number of relevant studies. We believe that it is necessary to delve deeper into research guided by current causal hypotheses about neural activity (Goswami, 2011, 2018) rather than studies focused on the behavioral symptoms manifested during reading. In this regard, some laboratories are making notable contributions with intracranial stimulation techniques, which are easier to apply than NF techniques (Marchesotti et al., 2020; Rufener et al., 2019).

For all these reasons, this review leads us to conclude that NF is not currently a robust treatment option to be applied with precision and safety in clinical contexts. However, there is no doubt that NF is a technique with potential, relatively inexpensive and non-invasive, and has shown its effectiveness in other developmental disorders. Therefore, NF could be of enormous utility in the rehabilitation of dyslexia, but it currently lacks theoretical and methodological rigor, making it crucial to use representative and well-selected samples, and, in general, rigorous experimental designs that allow for more precise and robust conclusions about the effectiveness of the treatments.

On the other hand, it is important to highlight that the diversity of Neurofeedback protocols applied in the reviewed studies, as well as the heterogeneity in therapeutic goals (attention, memory, language skills, etc.), makes it difficult to draw consistent conclusions about the efficacy of any single method. Although the protocol focused on reducing slow-wave activity (Theta/Delta) and increasing fast-wave activity (Beta) was the most frequently used and showed promising results, variations in experimental design, duration of interventions, and assessment instruments prevent it from being considered a clinically validated intervention. Therefore, we believe that future research should systematically compare the different existing protocols under methodologically controlled conditions, with larger samples and homogeneous inclusion criteria, in order to determine more precisely which intervention parameters are most effective in treating the core symptoms of dyslexia.

Beyond the methodological heterogeneity observed among the reviewed studies—such as differences in neurofeedback protocols, sample characteristics, and outcome measures—this review also faced notable challenges related to the accessibility and scientific quality of a substantial portion of the literature. Specifically, 95 potentially relevant records could not be retrieved due to factors including the absence of a DOI, the discontinuation of the publishing journals, or the unavailability of full texts despite repeated attempts. This situation, which reflects broader issues in the field of applied psychophysiology, underscores the lack of transparency and replicability in some research outputs. Moreover, several accessible studies presented methodological weaknesses, such as insufficient reporting, small sample sizes, or limited experimental control. These limitations not only constrain the generalizability of the findings but also point to an urgent need for more rigorous, standardized, and openly accessible research efforts in order to accurately assess the efficacy of neurofeedback interventions for developmental dyslexia.

## Supplementary Information

Below is the link to the electronic supplementary material.

Supplementary file1 (DOCX 29 KB)
