# Supplementary material for: Efficacy of neurofeedback in the treatment of Dyslexia: a systematic review
Source: Ann Dyslexia. 2025 Jul 17;76(1):137–55. doi: 10.1007/s11881-025-00335-0 (PMC13124931; doi:10.1007/s11881-025-00335-0)
Supplement: Supplementary file 1 — Supplementary file1 (DOCX 29 KB) [file 11881_2025_335_MOESM1_ESM.docx]

# Supplementary Table S1 – PRISMA Guidelines with Manuscript Locations

| PRISMA Guideline | Description | Location in Manuscript |
| --- | --- | --- |
| Eligibility criteria | Specify the inclusion and exclusion criteria for the review and how studies were grouped for the syntheses. | Method, Section 2.2 |
| Information sources | Specify all databases, registers, websites, organisations, reference lists and other sources searched or consulted to identify studies. Specify the date when each source was last searched or consulted. | Method, Section 2.5 |
| Search strategy | Present the full search strategies for all databases, registers and websites, including any filters and limits used. | Method, Section 2.2 |
| Selection process | Specify the methods used to decide whether a study met the inclusion criteria of the review, including how many reviewers screened each record and each report retrieved, whether they worked independently, and if applicable, details of automation tools used in the process. | Method, Section 2.5 |
| Data collection process | Specify the methods used to collect data from reports, including how many reviewers collected data from each report, whether they worked independently, any processes for obtaining or confirming data from study investigators, and if applicable, details of automation tools used in the process. | Method, Section 2.7 |
| Data items | List and define all outcomes for which data were sought. Specify whether all results that were compatible with each outcome domain in each study were sought (e.g. for all measures, time points, analyses), and if not, the methods used to decide which results to collect.  List and define all other variables for which data were sought (e.g. participant and intervention characteristics, funding sources). Describe any assumptions made about any missing or unclear information. | Methods, Section 2.8 |
| Study risk of bias assessment | Specify the methods used to assess risk of bias in the included studies, including details of the tool(s) used, how many reviewers assessed each study and whether they worked independently, and if applicable, details of automation tools used in the process. | Method, Section 2.6 |
| Effect measures | Specify for each outcome the effect measure(s) (e.g. risk ratio, mean difference) used in the synthesis or presentation of results. | Results |
| Synthesis methods | Describe the processes used to decide which studies were eligible for each synthesis (e.g. tabulating the study intervention characteristics and comparing against the planned groups for each synthesis (item #5)).  Describe any methods required to prepare the data for presentation or synthesis, such as handling of missing summary statistics, or data conversions.  Describe any methods used to tabulate or visually display results of individual studies and syntheses.  Describe any methods used to synthesize results and provide a rationale for the choice(s). If meta-analysis was performed, describe the model(s), method(s) to identify the presence and extent of statistical heterogeneity, and software package(s) used.  Describe any methods used to explore possible causes of heterogeneity among study results (e.g. subgroup analysis, meta-regression).  Describe any sensitivity analyses conducted to assess robustness of the synthesized results. | Results & Discussion |
| Reporting bias assessment | Describe any methods used to assess risk of bias due to missing results in a synthesis (arising from reporting biases). | Not applicable (no meta-analysis performed) |
| Certainty assessment | Describe any methods used to assess certainty (or confidence) in the body of evidence for an outcome. | Discussion, Limitations |
